# The Effect of Subjective Loss in Financial Risk Taking and Negative Emotion

**DOI:** 10.3389/fpsyg.2021.736353

**Published:** 2021-10-15

**Authors:** Dongmei Mei, Shasha He, Liman Man Wai Li, Yiyi Zhu

**Affiliations:** ^1^School of Psychology, Guizhou Normal University, Guiyang, China; ^2^Department of Psychology, The Education University of Hong Kong, Hong Kong, Hong Kong, SAR China; ^3^Department of Psychology, Sun Yat-sen University, Guangzhou, China

**Keywords:** subjective loss, risk-taking, financial decisions, regret, negative emotion

## Abstract

The current research examined the influence of subjective loss on financial risk-taking tendency and negative emotional experience through inducing the experience of subjective loss in auction scenarios. In Study 1, we found that the subjective loss experience (compared to no-loss experience) in an auction scenario induced greater financial risk propensity, especially in gambling, greater negative emotion, and greater decision regret. In addition, we found that the subjective loss experience induced stronger negative emotion but less risk propensity in investment than the actual loss experience did, but these two types of loss did not yield a difference in risk propensity in gambling in Study 2. These results implicate that subjective loss is a distinct experience from no-loss and actual loss experiences, which is reflected by the degree of associated emotional experience and subsequent risk-taking propensity. The current research highlights the complex psychological processes of the experience of loss in decision-making contexts.

## Introduction

There is a considerable interest, especially in research on decision making, in examining the influence of people’s prior decision-making experiences on the subsequent tasks. One well-studied prior experience is loss vs. gain. Studies have shown that losses generally loom larger than corresponding gains (Galanter and Pliner, 1974; Fishburn and Kochenberger, 1979) in the economic gain-loss framing (Sawers et al., 2011; Zhang et al., 2017; Kwak and Huettel, 2018). For instance, the negative feelings associated with a loss experience are generally desectorizing greater than those associated with a non-gain experience (Idson et al., 2000; Adjerid et al., 2021).

Taylor (1974) pointed out that loss can be in psychological/social terms or in functional/economic terms, or in some combinations of both forms, meaning that loss can be experienced because of an objective way (i.e., suffering from an actual loss) or a subjective way (i.e., missing the chance to obtain gains). In the current research, we argued that subjective loss, which is defined as the experience of missing chances to gain but not suffering from economic loss, would affect people’s financial risk propensity and negative emotional experiences, both are significant indicators of mental health as indicated by previous studies (Kircanski et al., 2018; Yakın et al., 2019; Kim and Ryu, 2021).

### Subjective Loss

The loss of resources causes stress, which makes individuals highly motivated to conserve their resources (Hobfoll, 1989; Hobfoll and Wells, 1998). Widespread evidence showed that people are more prone to avoid losses than acquire gains (Brooks and Zank, 2005; Seymour et al., 2015; Adjerid et al., 2021), which indicates a strong motivation for avoiding loss-related experiences. Usually, research on decision making focused on the effect of actual loss, which refers to the money lost when selling because an object’s value has decreased (Wilson, 2018). However, we may feel having losses when an actual loss does not occur in some situations. For example, people also experience negative emotions when they fail to grasp the opportunities in investing in promising projects or purchasing commodities with lower prices. Here, actual financial loss (e.g., money) does not occur, but negative feelings still arise. We named it as “subjective loss” in current research.

Despite the fact that a subjective loss does not consist of an actual economic loss, the psychological processes involved in this experience could be distinctive from one that contains no loss (in which people do not have loss objectively or subjectively). In fact, prior research findings converged to suggest that a subjective loss (i.e., missing opportunities) can be identified as a loss generally, which makes people motivated to prevent its occurrence (Fishburn and Kochenberger, 1979). For instance, people felt negative when they missed the opportunity to gain, though they actually lost nothing (Fishburn and Kochenberger, 1979; Bilgin, 2012; Beisswingert et al., 2015). Similarly, the research of portfolio management demonstrated that, while the decision-maker aims at protecting portfolio wealth from downside risk (prevention of actual loss), he/she is also reluctant to over-protect the portfolio because this may potentially increase the chance of missing gaining opportunities (prevention of subjective loss) (Kawas and Thiele, 2011; Adjerid et al., 2021). Taken together, decision-makers indeed care about actual loss as well as subjective loss. Based on these findings, we proposed that the subjective loss experience can be regarded as a kind of loss, which may induce similar influence as the actual loss experience does in different domains, including risk taking and negative emotional experiences.

### Risk Taking in Investment and Gambling Following Loss

People are generally risk-averse. However, when an individual is in a state of loss, risk seeking becomes a motivational necessity (Scholer et al., 2010; Seymour et al., 2015; Herman et al., 2018), meaning that the loss state motivates people to compensate for the previous loss by taking more risks in the subsequent opportunities. In other words, people become more risk-taking following prior loss experience. Some indirect evidence from emotional research indicated that negative experiences (e.g., losses) promoted risk taking (Schneider et al., 2016; Ferrer et al., 2017; Herman et al., 2018; Zou et al., 2019). Similarly, research on decision making showed that following potential losses, individuals’ risk-taking propensity increased in the subsequent decision-making tasks (Weber and Zuchel, 2005; Tom et al., 2007). Staw (1976) argued that people tended to stick to a course of action more likely (i.e., escalated commitment) and consequently take greater risk after having a loss than a gain. According to the framing effect in prospect theory (Kahneman and Tversky, 1979; Schneider et al., 2016; Ruggeri et al., 2020), the risk preference vary depends on whether a choice is made in terms of gains or losses, even when the prospects of the options are held constant. Participants immersed in a miss scenario (i.e., did not take an action) may overestimate the likelihood of gain and thus take more risks in subsequent decisions (McDermott, 2004). In the framework of organizational decision making, opportunity cost may increase sustaining commitment in a risky investment (Northcraft and Neale, 1986). Therefore, we speculate that experiencing a subjective loss, potentially a loss-framed message, may also influence the risk propensity of decision-makers in subsequent decisions. With the assumption that subjective loss could be experienced as one type of loss, we expected that a subjective loss experience, like an actual loss experience, would promote greater risk taking (as compared with a no-loss experience).

Risk taking is domain-specific (Weber et al., 2002; Ozorio and Fong, 2004; Shou and Olney, 2020), which means that the risk-taking tendency in one domain is not expected to be highly associated with that in other domains. In the current research, we examined the effect of subjective loss in two risk-taking behaviors in the financial domain: Investment vs. Gambling. Investment vehicles such as mutual funds and blue-chip stocks tend to entail low risk and are typically held for a period of months or years (Arthur et al., 2016). In contrast, most forms of gambling are at high risk of losing one’s stake, and the outcome is typically known within just seconds (e.g., Scratch tickets and electronic gambling machines), minutes (e.g., bingo, horse racing, and keno), or days (e.g., lotteries and sports betting). These two financial decisions have been found to be conceptually different (Weber et al., 2002; Arthur et al., 2016). Specifically, investment was found to be related to the achievement of goals (consequential utility) while gambling was found to be related to immediate sensations and excitement (process utility) (Le Menestrel, 2001; Ozorio and Fong, 2004). Similarly, it was found that gambling activities (vs. investment activities) were associated more strongly with impulsivity and emotional instability, and especially with high risk tolerance (Arthur et al., 2016). These findings seemed to suggest that gambling would be more vulnerable to the impact of the subjective (or emotional) state compared with investment. Therefore, we expected that a subjective loss, an experience with subjective nature, as compared with a no-loss experience, would increase risk taking in gambling whereas the effect of subjective loss would be less obvious in investment.

### Negative Emotion Following Losses

In addition to risk taking, we also explored the effect of subjective loss on emotional experiences, which are essential factors in influencing people’s decision-making processes (Lerner and Keltner, 2001; Beisswingert et al., 2015; Lerner et al., 2015). We focused on the overall negative emotion and decision regret in the current research.

The negative outcomes, such as losses, usually bring negative feelings (e.g., sad and regret) about their decisions, or even about themselves (Lerner and Keltner, 2000, 2001; Chua et al., 2009; Seymour et al., 2015). According to the disappointment theory (Bell, 1985; Kugler et al., 2012; Bench et al., 2021) and other related theories (Van Dijk and Zeelenberg, 2005; Lench and Bench, 2015), negative emotion (e.g., disappointment) usually results from receiving an outcome that is worse than expected.

Of different negative emotions, regret is a complex and counterfactual emotion (Gilovich et al., 2003; Pham, 2007), which has received the most research attention from decision theorists (Connolly and Zeelenberg, 2002). Regret can be both an antecedent and a consequence of decision making (Connolly and Zeelenberg, 2002; Bench et al., 2021). The counterfactual thoughts related to the decision maker’s own behavior and actions are associated with regret (Van Dijk and Zeelenberg, 2005). Therefore, regret can be easily experienced when we make a comparison between an outcome and an outcome foregone. Economic choice theorists point out that people would feel regret if a decision outcome was worse than what they would have received by choosing a different option (Loomes and Sugden, 1982). Consistently, some research showed that the regret was higher when the outcomes of the alternative forgone were better than the outcomes of the alternative selected (Pachur et al., 2018; Zahera and Bansal, 2018).

According to the abovementioned theories, the regret feeling in the subjective loss scenario is associated with the comparative evaluation of the outcome. Thus, we speculated that participants experiencing a missed-opportunity scenario would have stronger negative emotions and feel more decision regret than participants experiencing a no-loss scenario. However, whether a subjective loss experience would induce greater negative emotion (or decision regret feeling) than an actual loss experience was unknown.

## Overview of Current Research

The primary goal of the current research was to examine the effects of subjective loss on risk taking and negative emotion, respectively. We hypothesized that a subjective loss (vs. no loss) in a prior decision-making task would induce stronger negative emotion and greater risk propensity in the financial domain, especially in gambling. To test it, we manipulated loss experiences (no-loss vs. subjective loss) in auction scenarios in Study 1 to test whether the subjective loss experience would be distinctive from the no-loss experience. In Study 2, we included an actual loss condition to compare with the effect of a subjective loss. We explored whether the subject loss would differ from the actual loss in terms of the influences on financial risk propensity and negative emotion.

## Study 1

We examined the effects of subjective loss on the financial risk-taking tendency and negative emotion. We hypothesized that, as compared with no-loss experience, subjective loss experience would induce greater financial risk propensity, especially in gambling, and greater negative emotion, including decision regret.

### Materials and Methods

#### Participants

We recruited 98 undergraduates (65 females, mean age: 20.49 years, *SD* = 1.52) for the experiment in exchange for partial course credits. The sample size was determined by referring to the number of participants in previous studies on decision making (Slattery and Ganster, 2002; Anderson and Galinsky, 2006; Zhang et al., 2017; Kwak and Huettel, 2018). The study was approved by the departmental research ethics committee from a university in China. The consent was given by the participants before they participated in the study.

#### Materials and Procedure

Based on the prospect theory (Kahneman and Tversky, 1979; Ruggeri et al., 2020), to provide a context for risk decision, we included the predicted price and the actual value of the commodity in the auction scenarios. Through the auction scenarios, participants could experience the missed opportunities (subjective loss), cost-effective experience (no-loss), as well as an actual loss for money.

##### Decision-Making Scenario

First, participants were randomly assigned to read either a subjective loss (*n* = 51) or no-loss (*n* = 47) auction scenario. In the subjective loss scenario, participants were told that they missed the bid of commodity, in which they actually lost nothing. In the no-loss scenario, participants did not have any loss-related experiences (see Appendix for the auction scenarios).

To check their comprehension of the auction scenario, participants were asked to answer some questions regarding the auction scenario. We set up four questions to check whether participants paid attention and fully understood the scenario. The questions were, “*Did you successfully get the commodity you liked in the auction scenario?*,” “*What was the final sale price of your favorite commodity in the auction scenario?*,” “*What was your expected price for the commodity you liked in the auction scenario?*,” and “*What was the actual price of the commodity you liked in the auction scenario?*” Participants who had answered these four items correctly were considered as passing the attention check and fully understanding the scenario. Twenty-six participants were excluded because they did not fully understand the materials.

To check whether the scenario elicited the participant’s subjective loss feeling, participants were asked “*How much money do you think you lose/missed in the scenario situation?*” after completing the attentional and comprehension measures. The responses were recoded as 1 (loss or miss) and 0 (no loss or miss) according to participants’ answers. The higher the probability of the loss response, the more the participants in a given condition subjectively perceived the loss.

##### Measures of Emotion

Next, participants answered questions related to their emotional experiences. We used two measures to assess participants’ negative emotions. First, we measured participants’ general negative emotions. Participants rated their emotional intensity for several emotions (modified from PANAS) (Watson et al., 1988) using a five-point Likert scale (1: very slightly or not at all; 5: extremely). The reliabilities were acceptable [positive affection (PA): Cronbach’s alpha = 0.884; negative affection (NA): Cronbach’s alpha = 0.746]. We averaged scores of the items for PA and NA, respectively. PA was further subtracted from NA to calculate an index of general negative emotion (NA-PA) with higher scores indicating greater intensity in negative emotions (Haslam et al., 2009).

Among different negative emotions, regret is frequently studied in decision-making research (Chua et al., 2009). Therefore, we also measured participants’ decision regret, the second indicator of negative emotion in the current research. Participants reported their decision regret by completing the Decision Regret Scale (Brehaut et al., 2003) with a five-point Likert scale range from 1 (strongly agree) to 5 (strongly disagree). The sample items include, “*It was the right decision*” and “*I regret the choice that was made (reverse scoring)*.” The reliability was satisfactory (Cronbach’s alpha = 0.823). An average score of all items was computed with higher scores indicating a higher level of regret in the described decision-making scenario.

##### Financial Risk-Taking Scale

At last, participants reported their financial risk propensity, in which the items were extracted from the Domain-Specific Risk-taking (adult) Scale (Blais and Weber, 2006). The scale is wildly used as a measurement of dependent variable in research on risk propensity (Ben-Zur and Zeidner, 2009; Devlin et al., 2015). Importantly, recent studies demonstrated that the risk-taking rating of this scale can be sensitive in reflecting the condition of manipulations (Tymula et al., 2012; Li et al., 2016, 2019; Farnham et al., 2018; Shou and Olney, 2020). Therefore, we considered this scale as a meaningful tool to detect the effect of manipulation in the current research. Participants rated their agreement on each item using a seven-point Likert scale ranging from 1 (extremely unlikely) to 7 (extremely likely). Two sample items include, “*Investing 10% of your annual income in a moderate growth mutual fund*” (investment) and “*Betting a day’s income at the horse races”* (gambling). The reliabilities of the overall Financial risk-taking scale (Cronbach’s alpha = 0.738), Investment risk-taking subscale (Cronbach’s alpha =0.662), and the Gambling risk-taking subscale (Cronbach’s alpha = 0.646) were acceptable. Thus, average scores of all items for the overall financial risk propensity, risk propensity in investment, and risk propensity in gambling were computed separately, with higher scores indicating greater risk propensity.

At the end of the study, all participants were fully debriefed and thanked for their participation after answering few demographic questions.

### Results

Before conducting the major analyses, we first explored the effect of gender and age because several previous studies has revealed significant gender and age effects on risk-taking tendency (Charness and Gneezy, 2012; Perryman et al., 2016; Xie et al., 2017). Through independent *t*-test analysis, the effect of gender on negative emotions [*t*(70) = 0.18, *p* = 0.86, Cohen’s *d* = 0.04, 95% CI (–0.49, 0.59)], decision regret [*t*(70) = 0.29, *p* = 0.77, Cohen’s *d* = 0.07, 95% CI [–0.33, 0.45)], financial risk propensity [*t*(70) = 1.35, *p* = 0.18, Cohen’s *d* = 0.31, 95% CI [–0.17, 0.89)], the risk propensity in investment [*t*(70) = 1.05, *p* = 0.30, Cohen’s *d* = 0.24, 95% CI [–0.31, 0.98)], or the risk propensity in gambling [*t*(70) = 1.26, *p* = 0.21, Cohen’s *d* = 0.29, 95% CI [–0.22, 0.98)] was not significant. The effect of age was also not significant for negative emotion (*r* = 0.08, *p* = 0.51), decision regret (*r* = 0.12, *p* = 0.33), financial risk propensity (*r* = 0.17, *p* = 0.17), the risk propensity in investment (*r* = 0.16, *p* = 0.18), and the risk propensity in gambling (*r* = 0.12, *p* = 0.31). The non-significant results were consistent with recent meta-analyses and empirical studies that have not been able to verify the effects forcefully (Nelson, 2015; Friedl et al., 2020). In addition, all results remained similar when we controlled for the effects associated with gender and age. Thus we collapsed the data for the final analyses in both studies.

#### Manipulation Check

The Chi-square test analysis showed that a greater proportion of participants in the subjective loss condition (90%) reported having loss or missing money than the participants in the no-loss condition (23.81%), χχ^2^(1) = 30.69, *p* < 0.001. The results illustrated that the manipulation of subjective loss was successful.

#### Negative Emotion

**FIGURE 1:**
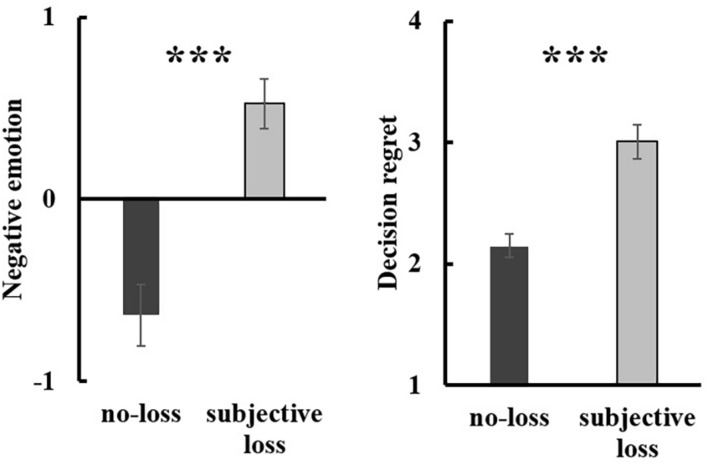
The loss effect on general negative emotion (left) and decision regret (right) in Study 1. Error bars represent standard errors. ***p < 0.001.

An independent *t*-test analysis revealed a significant group difference in general negative emotion, [*t*(70) = –5.03, *p* < 0.001, Cohen’s *d* = –1.24, 95% CI (–1.62, –0.70)]. Consistent with our hypothesis, participants with a subjective loss experience (*M* = 0.52, *SD* = 0.76) reported greater negative emotion relative to those with a no-loss experience (*M* = –0.64, *SD* = 1.09) (see Figure 1). For decision regret, consistently, participants in the subjective loss condition (*M* = 3.01, *SD* = 0.77) reported greater decision regret relative to those in the no-loss condition (*M* = 2.15, *SD* = 0.62), [*t*(70) = –5.18, *p* < 0.001, Cohen’s *d* = –1.21, 95% CI (–1.18, –0.53)] (see Figure 1).

#### Financial Risk Propensity

**FIGURE 2:**
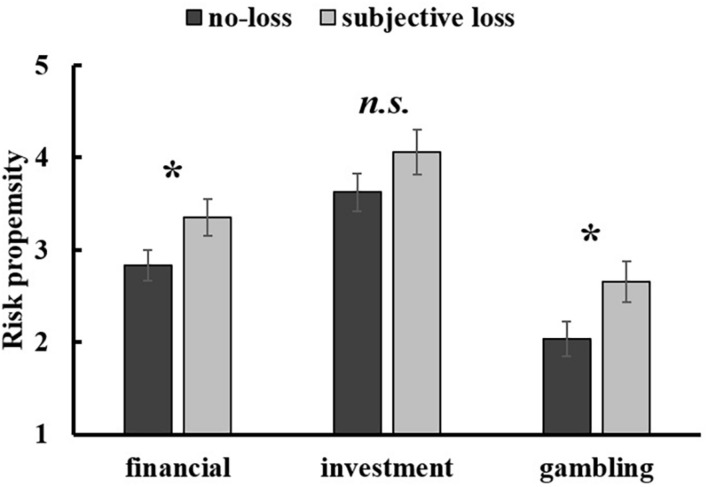
Figure 2. The loss effect on the overall financial risk propensity, risk propensity in investment, and risk propensity in gambling in Study 1. Error bars represent standard errors. n.s. *p* > 0.05, * *p* < 0.05.

The analyses of loss effect on financial risk-taking by independent *t*-test analysis showed that participants in the subjective loss condition (*M* = 3.36, *SD* = 1.07) were more risk-taking than participants in the no-loss condition (*M* = 2.83, *SD* = 1.10), [*t*(70) = –2.03, *p* = 0.046, Cohen’s *d* = –0.49, 95% CI (–1.04, –0.01)] (see Figure 2).

To investigate the effect of subjective loss in risk propensity in investment and gambling, a single factor MANOVA was performed. The results showed that the risk propensity in investment was not significantly different between the subjective loss condition (*M* = 4.06, *SD* = 1.22) and the no-loss condition (*M* = 3.63, *SD* = 1.40), [*F*(1, 70) = 1.83, *p* = 0.181, $\eta_{p}^{2}$ = 0.025], whereas the risk propensity in gambling was significant higher in the subjective loss condition (*M* = 2.66, *SD* = 1.25) than in the no-loss condition (*M* = 2.03, *SD* = 1.21), [*F*(1, 70) = 4.52, *p* = 0.037, $\eta_{p}^{2}$ = 0.061] (see Figure 2).

Table 1 presents the intercorrelations among the measured variables. Negative emotion was not significantly associated with financial risk propensity (*r* = 0.065, *p* = 0.589), risk propensity in investment (*r* = 0.095, *p* = 0.425), or risk propensity in gambling (*r* = 0.113, *p* = 0.914). Decision regret was also not significantly associated with financial risk propensity (*r* = –0.001, *p* = 0.992), risk propensity in investment (*r* = 0.063, *p* = 0.601), or risk propensity in gambling (*r* = –0.069, *p* = 0.567).

**TABLE 1:** Correlation analysis of key measured variables in Studies 1 and 2.

	Study 1 (*n* = 72)				Study 2 (*n* = 59)			
	1	2	3	4	1	2	3	4
1. Negative emotion	–				–			
2. Decision regret	0.633**	–			0.621**	–		
3. Financial risk propensity	0.065	–0.001	–		–0.352**	–0.104	–	
4. Risk propensity in investment	0.095	0.063	0.865**	–	–0.409**	–0.176	0.805**	–
5. Risk propensity in gambling	0.013	–0.069	0.847**	0.466**	–0.167	0.004	0.819**	0.319*

* p < 0.05, ** p < 0.01.

### Discussion

We found that a subjective loss experience increased financial risk propensity, especially in gambling, as compared to a no-loss experience. In addition, the subjective loss experience also induced greater negative emotion (indicated by both general negative emotion and decision regret) than the no-loss experience. These results indicated that the subjective loss experience was different from neutral (i.e., no loss) experience. Although we found that there were differences in risk taking and negative emotion between subjective loss experience and no-loss experience, the question of whether the effect of subjective loss experience would differ from that of actual loss experience was still unknown. To address this issue, we included an actual loss condition to better understand the influence of subjective loss in Study 2. Moreover, the high exclusion of data was a major limitation identified in Study 1. To enhance participants’ comprehension of the study material, we have revised the experimental procedure in Study 2.

## Study 2

As discussed in the introduction, we assumed that subjective loss could be understood as a type of loss, even though there are no actual losses. However, it was unknown whether the subjective and actual loss would differ in terms of induced financial risk propensity and negative emotion. We explored this question in Study 2.

### Materials and Methods

#### Participants

We recruited 70 undergraduates (21 females, mean age: 21.89 years, *SD* = 1.89) to participate in our experiment in exchange for partial course credits. Further data collection was not feasible because of the end of the academic year.

#### Materials and Procedure

Instead of having a no-loss condition, we included an actual loss condition in Study 2. Participants were randomly assigned to either an actual loss or a subjective loss condition, and each condition included 35 participants. In the actual loss condition, participants got the product with a price that was higher than the real market price of the product (see Appendix). The auction scenario in the subjective loss condition was the same used in Study 1.

In consideration of the high exclusion rate in study 1, we have modified the attentional and comprehension measures in Study 2. The auction scenario was presented twice. In the second presentation, the expected price, transaction price, and actual value of the commodity in the scenario were left blank, and participants needed to fill in the information. Only participants who provided correct answers were considered as passing the attention check and fully understanding the scenario. Thus, the data of 11 participants were excluded.

Similar to Study 1, participant’s loss feelings were measured after completing the attention and comprehension tests. And 100% of participants reported having loss or missing money feelings in both conditions, which illustrated that both subjective and actual loss induced a sense of loss.

The scales measured were the same as Study 1. The reliabilities of all used scales were satisfactory (PA: Cronbach’s alpha = 0.843; NA: Cronbach’s alpha = 0.839; Decision Regret Scale: Cronbach’s alpha = 0.704; Financial risk taking scale: Cronbach’s alpha = 0.697; investment risk taking subscale: Cronbach’s alpha = 0.634; gambling risk taking subscale: Cronbach’s alpha = 0.704).

### Results

Like Study 1, the effects of gender and age were also examined. Consistent with Study 1, we did not find a significant effect of gender on negative emotion [*t*(57) = 0.64, *p* = 0.53, Cohen’s *d* = 0.19, 95% CI (–0.56, 1.09)], decision regret [*t*(57) = 1.36, *p* = 0.18, Cohen’s *d* = 0.41, 95% CI (–0.11, 0.59)], financial risk propensity [*t*(57) = 0.33, *p* = 0.74, Cohen’s *d* = –0.10, 95% CI [–0.68, 0.49)], the risk propensity in investment [*t*(57) = –0.61, *p* = 0.55, Cohen’s *d* = –0.17, 95% CI [–0.92, 0.49)], or the risk propensity in gambling [*t*(57) = 0.05, *p* = 0.96, Cohen’s *d* = 0.02, 95% CI [–0.71, 0.75]). And we did not find a significant age effect for negative emotion (*r* = –0.05, *p* = 0.70), financial risk propensity (*r* = 0.06, *p* = 0.66), the risk propensity in investment (*r* = 0.12, *p* = 0.35), and the risk propensity in gambling (*r* = –0.03, *p* = 0.85). However, the correlation between age and decision regret was significant (*r* = –0.29, *p* = 0.03). But all results remained similar when we controlled for the effects associated with gender and age.

#### Negative Emotion

**FIGURE 3:**
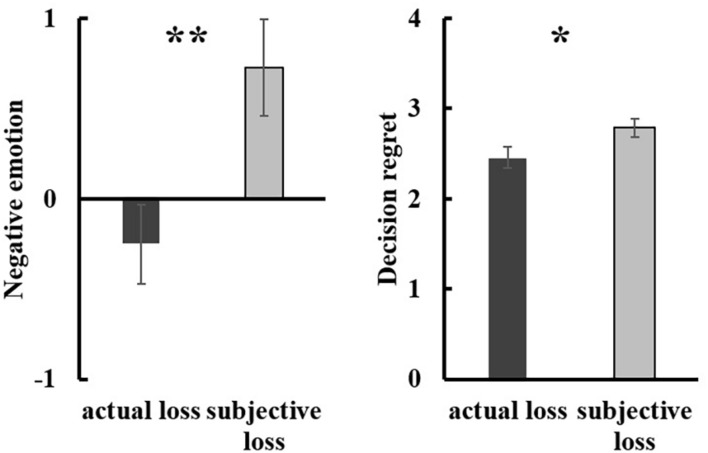
The loss effect on general negative emotion (left) and decision regret (right) in Study 2. Error bars represent standard errors. *p < 0.05; **p < 0.01.

The findings revealed a significant group difference in general negative emotion, [*t*(57) = 2.78, *p* = 0.007, Cohen’s *d* = 0.72, 95% CI (0.27, 1.69)]. Participants in the subjective loss condition (*M* = 0.73, *SD* = 1.47) reported greater negative emotion than those in the actual loss condition did (*M* = –0.25, *SD* = 1.24) (see Figure 3). In addition, participants in the subjective loss condition (*M* = 2.79, *SD* = 0.53) reported more decision regret than those in the actual loss condition did (*M* = 2.46, *SD* = 0.65), [*t*(57) = 2.10, *p* = 0.040, Cohen’s *d* = 0.56, 95% CI [0.02, 0.64] (see Figure 3).

#### Financial Risk Propensity

**FIGURE 4:**
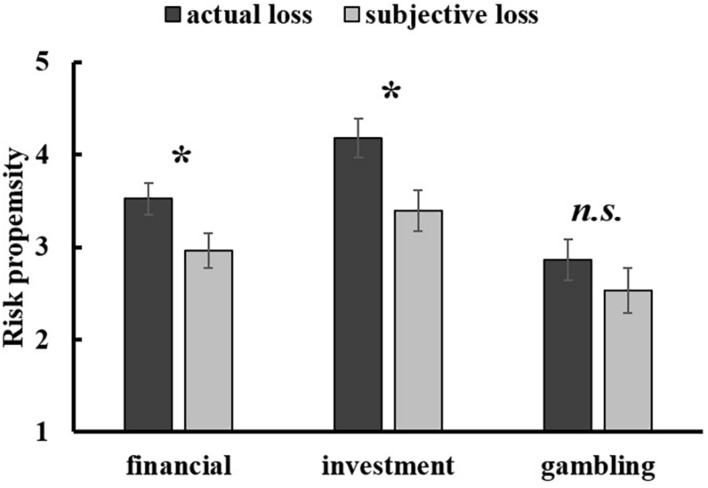
The loss effect on the overall financial risk propensity, risk propensity in investment, and risk propensity in gambling in Study 2. Error bars represent standard errors. n.s. *p* > 0.05; *p < 0.05.

The analyses of loss effect on financial risk propensity by independent *t-*test analysis showed that participants in the subjective loss condition (*M* = 2.96, *SD* = 0.86) were less risk-taking than participants in the actual loss condition (*M* = 3.52, *SD* = 1.07), [*t*(57) = –2.19, *p* = 0.033, Cohen’s *d* = –0.58, 95% CI [–1.07, –0.05] (see Figure 4).

The results showed that participants became more risk-taking in investment in the actual loss condition (*M* = 4.18, *SD* = 1.30) than in the subjective loss condition (*M* = 3.40, *SD* = 0.98), [*F*(1, 57) = 6.60, *p* = 0.013, $\eta_{p}^{2}$ = 0.104; however, the risk propensity in gambling did not differ between the actual loss condition (*M* = 2.86, *SD* = 1.34) and the subjective loss condition (*M* = 2.53, *SD* = 1.16), [*F*(1, 57) = 1.03, *p* = 0.315, $\eta_{p}^{2}$ = 0.018 (see Figure 4).

Negative emotion was significantly associated with financial risk propensity (*r* = –0.352, *p* = 0.006) and risk propensity in investment (*r* = –0.409, *p* = 0.001), but it was not significantly associated with risk propensity in gambling (*r* = –0.167, *p* = 0.206). Decision regret was not significantly associated with financial risk propensity (*r* = –0.104, *p* = 0.433), risk propensity in investment (*r* = –0.176, *p* = 0.183), and risk propensity in gambling (*r* = 0.004, *p* = 0.976; see Table 1).

### Discussion

We explored and compared the influence of subjective loss and actual loss on people’s risk propensity in the financial domain and negative emotion in Study 2. We found that the subjective loss experience induced greater negative emotions (both general negative emotion and decision regret) but less risk taking in the financial domain (especially in investment) than the actual loss experience. These results suggested that subjective loss experiences were not identical to actual loss experiences. We further discussed these findings in the general discussion.

## General Discussion

The current research examined the effect of subjective loss in negative emotion and the financial risk-taking propensity. In Study 1, we found that a subjective loss experience made people feel stronger negative emotions and more risk-taking in the financial domain (especially in gambling) than a no-loss experience. In Study 2, we found that participants who experienced subjective loss showed less risk propensity (especially in investment) but greater negative emotion than those who experienced actual loss. Combining the findings from these two studies, we found that the subjective loss experience was different from neutral experiences, which was indicated by its stronger effect in inducing negative emotion and financial risk propensity, especially in gambling. Yet subjective loss experience was not identical to actual loss experience, which was indicated by its stronger effect inducing negative emotion but a weaker effect in inducing financial risk propensity, especially in investment.

The current research was consistent with multiple prior studies emphasizing that risk taking increases after experiencing financial losses (Idson et al., 2000; Weber and Zuchel, 2005; Schneider et al., 2016). Loss experience leads to an escalation of commitment, which, in turn, leads to greater risk taking in subsequent decision-making tasks. More importantly, the current research showed that the subjective sense of having loss is strong enough to promote financial risk propensity (Study 1), although its overall effect in risk taking is not comparable to an actual loss (Study 2). Taken together, consistent with previous research (Bilgin, 2012), these findings illustrated that subjective loss has an important influence during decision-making processes. Besides, we found that subjective loss had a stronger influence on gambling as compared with no loss, whereas its influence on gambling did not differ from the actual loss. Taken together, it suggested that subjective loss can affect risk taking particularly in stimulating situations like gambling, which may also indicate that subjective loss has a stronger effect on the decisions that are more vulnerable to subjective/psychological activities.

Regarding the emotional experience, we obtained evidence showing that the subjective loss induced greater negative emotion and decision regret compared with no loss and an actual loss. Consistent with previous theories and research (Bell, 1985; Van Dijk and Zeelenberg, 2005; Kugler et al., 2012; Lench and Bench, 2015; Bench et al., 2021), our results demonstrated that subjective loss can cause people to have negative emotions and regret just as actual loss scenarios do. It is interesting to note that people reported intense negative emotion and decision regret after missing opportunities to gain. Relatedly, we found that negative emotion was more strongly correlated with people’s financial risk propensity (especially in investment) in Study 2 than in Study 1 (see Table 1), which indicated that the negative feeling, both elicited by subjective and objective loss experience, would impact the subsequent risk propensity more strongly than that in the no-loss experience. Moreover, our results showed that, compared with the objective loss scenario, the subjective loss scenario led to stronger negative emotion and feelings of regret. This result was consistent with recent evidence which showed that people experience higher levels of regret when the outcomes of the forgone alternative were better than the outcomes of the selected alternative (Pachur et al., 2018; Zahera and Bansal, 2018). The reason could be due to inaction in the subjective loss experience (Zeelenberg et al., 2002), in which inaction may generate a great amount of long-term negative emotion because of frequent rumination of missing opportunities (Gilovich et al., 2003). These findings suggest that the negative emotions caused by subjective loss can be more profound, which has been neglected in previous research on decision making.

### Implications

The current research found that both kinds of loss, either subjective loss or actual loss, promoted subsequent financial risk propensity. Not surprisingly, people are sensitive to an actual economic loss. As evidenced by economic research, the loss experience motivates people to be risk-taking in the portfolio decision circumstances (Weber and Zuchel, 2005; Herman et al., 2018; Bench et al., 2021). Economic research in decision making primarily focuses on the influence of actual loss (Collins and Lapsley, 2003; Beisswingert et al., 2015; Ferrer et al., 2017; Kwak and Huettel, 2018) although economists acknowledged that subjective loss can shape our psychological processes. Supporting this notion, the current research found that the subjective sense of loss is strong enough to affect people’s decision-making processes. Thus, our research extended the economic research from solely focusing on the influence of actual loss to explore the influence of subjective loss that was not well-studied in the prior economic research.

The results of this research further underscore the importance of psychological processes (or subjective experiences) in decision-making processes. As Simon (1960) has emphasized, people are not always rational for decision making. Instead, a lot of humanistic characters, such as motivation and affective states, affect decision making. The importance of these humanistic characters should be emphasized in future consumer research. Extending the current findings, we can explore how subjective loss experience that involves intense psychological processes would affect consumers’ purchasing behaviors, which can be greatly affected by negative affective states (Han et al., 2007; Zou et al., 2019). For example, future research can examine whether creating a sense of potential subjective loss, such as telling the consumers that they may miss the chance to get the product at a lower price later, would increase their subsequent consumption.

In addition, the current research found that subjective loss experience induced significantly greater negative emotions like regret and sadness about the prior decision. These findings indicated a strong influence of subjective/emotional states in decision making. Future research should continue to examine the role of emotional experiences in different decision-making processes. For example, future research can investigate whether emotional experiences, which may be informative, can directly shape negotiation processes. The induced emotion provides background information that can serve as input for negotiators’ strategic decision making (Van Kleef et al., 2004; Lerner et al., 2015). It also serves a social function, intentionally directing the recipients to know the status of the interpersonal relationships and the sender’s social intentions (Fridlund, 1992; Knutson, 1996). Future research should thoroughly investigate how the induced emotion can potentially affect the negotiation processes and the outcomes.

### Limitations

There were some limitations, which could attribute to a weaker effect size observed in the current research. Regardless, smaller effect sizes should not be neglected in psychological science (Götz et al., 2021).

One limitation was that all participants were from China, where the concept of “loss” may be more salient due to the prevalence of prevention focus (Li and Masuda, 2015; Adjerid et al., 2021). It would be important to replicate the obtained findings in other societies that have a strong promotion-focus orientation, such as America and Canada.

Other limitations were related to the design of the research. First, we compared subjective loss experience with no-loss experience and actual loss experience separately in the two studies. The difference in the design of the two studies in the current research did not allow us to collapse the responses for this purpose. To fully understand the differences among these three types of experiences, we should have a study design with three conditions (no loss, actual loss, and subjective loss) in future research.

In addition, the adopted scenarios might have induced more complex experiences than we expected. For instance, we found that participants in the actual loss condition experienced neutral emotion instead of negative emotion, which might be because they have obtaining the desired object compensated the experience of actual loss. The complex experiences may weaken the expected effect size. Therefore, future studies need to control for confounds more carefully.

These complex experiences might also explain the higher number of participants excluded in the two studies despite our attempt of improving the design in Study 2. Another reason for the higher exclusion rate could be due to the fact that the loss is an abstract conception (Isen et al., 1988). To test our hypotheses, participants needed to experience subjective loss or actual loss as planned in the auction scenario, so understanding the scenario correctly was the first prerequisite. Although the questions we set were straightforward in Study 1, some participants did not fully understand the situation. Intending to enhance the participants’ comprehension, we presented the auction situation repeatedly in Study 2. However, the high exclusion rate still existed. We speculated that it could be because the loss, especially the subjective loss, is an abstract conception (Isen et al., 1988). More studies are needed to better understand the nature of loss, especially for subjective loss, which is crucial for scenarios that can easily trigger participants’ subjective loss experiences.

Considering the previous evidence of gender effect in risk taking (Charness and Gneezy, 2012; Perryman et al., 2016; Xie et al., 2017), the unbalanced distribution of gender across conditions in the current research may affect the stability of our results. However, other studies showed that the effect of gender on risk taking was not notable (Nelson, 2015; Friedl et al., 2020), which may suggest that this concern is minimal.

Moreover, the actual loss and subjective loss experiences manipulated by scenarios were both hypothetical in the current study; it would be better to examine the actual experiences. Additionally, to check the robustness of the current findings, the self-reported measures need to be extended. Future studies need to measure physiological responses or actual behaviors as dependent variables.

Finally, although we found that the subjective loss affected our decision-making processes, the underlying mechanisms of how it can promote risk taking should be examined in future research.

## Conclusion

The current research found that subjective loss was an independent state differing from no loss and actual loss. The subjective loss that may involve more complicated psychological processes induced stronger negative emotion than the no-loss experience and actual loss experience. The current research implicates the importance of understanding the psychological processes (subjective experiences) in decision-making processes.

## Data Availability Statement

The original contributions presented in the study are included in the article/supplementary material, further inquiries can be directed to the corresponding author/s.

## Ethics Statement

The studies involving human participants were reviewed and approved by the Department of Psychology, Sun Yat-sen University. The patients/participants provided their written informed consent to participate in this study.

## Author Contributions

DM conceptualized the study, collected the data, conducted statistical analyses, drafted the manuscript, and revised and prepared the manuscript for submission. LL contributed to the conceptualization of the study, supervision, and revision of the manuscript. SH and YZ aided with statistical analyses and revised the manuscript. All authors contributed to the article and approved the submitted version.

## Conflict of Interest

The authors declare that the research was conducted in the absence of any commercial or financial relationships that could be construed as a potential conflict of interest.

## Publisher’s Note

All claims expressed in this article are solely those of the authors and do not necessarily represent those of their affiliated organizations, or those of the publisher, the editors and the reviewers. Any product that may be evaluated in this article, or claim that may be made by its manufacturer, is not guaranteed or endorsed by the publisher.

## Appendix

The study material was presented in Chinese (Chinese material is available online: https://osf.io/v4uqb/?view_only=e69a02584bda4b9b90d857cdc8939cf0).

The scenario of subjective loss condition (in Studies 1 and 2) is as follow:

*Last week, you attended an auction activity with your friends. In that activity, there were different kinds of commodities; among them, there were some commodities that you liked. At the beginning of the auction, you were really interested in one commodity, the initial price was 200 yuan and your expected price was 1000 yuan.*

*You said to yourself, if the price was higher than 1000 yuan, you would give up. The auction started and the commodities were put on the table, people started to shout out their competitive price. The price was getting higher and reached 800 yuan quickly. At this time, you were worried about missing this commodity, so you were going to shout out your expected price, but someone else had already beaten you to it, “1000 yuan!.” The auctioneer shouted out sequentially, “1000 yuan for the first time!,” “1000 yuan for the second time!,” “1000 yuan for the third time!.” When you were hesitant, the auctioneer announced a deal! Someone who spent 1000 yuan finally got the commodity.*

*A week later, today, one of your knowledgeable friends told you that the real price of the commodity you missed in the auction is 1500 yuan.*

The no-loss condition was similar to the auction scenario described in the subjective loss condition except that the participant (i.e., you) successfully bided the commodity by 1000 yuan with its same object value.

The scenario of no-loss condition (in Study 1) is as follow:

*Last week, you attended an auction activity with your friends. In that activity, there were different kinds of commodities; among them, there were some commodities that you liked. At the beginning of the auction, you were really interested in one commodity, the initial price was 200 yuan and your expected price was 1000 yuan.*

*You said to yourself, if the price was higher than 1000 yuan, you would give up. The auction started and the commodities were put on the table, people started to shout out their competitive price. The price was getting higher and reached 800 yuan quickly. At this time, you were worried about missing this commodity, so you shout out your expected price, “1000 yuan!”. The auctioneer shouted out sequentially, “1000 yuan for the first time!,” “1000 yuan for the second time!,” “1000 yuan for the third time!.” You spent 1000 yuan finally got the commodity.*

*A week later, today, one of your knowledgeable friends told you that the real price of the commodity you got in the auction is 1000 yuan.*

The actual loss condition (Study 2) was similar to the auction scenario described in the subjective loss condition except that the participant (i.e., you) successfully bided the commodity by 1000 yuan with its object value only 500 yuan.

The scenario of actual loss condition (in Study 2) is as follow:

*Last week, you attended an auction activity with your friends. In that activity, there were different kinds of commodities; among them, there were some commodities that you liked. At the beginning of the auction, you were really interested in one commodity, the initial price was 200 yuan and your expected price was 1000 yuan.*

*You said to yourself, if the price was higher than 1000 yuan, you would give up. The auction started and the commodities were put on the table, people started to shout out their competitive price. The price was getting higher and reached 800 yuan quickly. At this time, you were worried about missing this commodity, so you shout out your expected price, “1000 yuan!.” The auctioneer shouted out sequentially, “1000 yuan for the first time!,” “1000 yuan for the second time!,” “1000 yuan for the third time!.” You spent 1000 yuan finally got the commodity.*

*A week later, today, one of your knowledgeable friends told you that the real price of the commodity you got in the auction is 500 yuan.*
